# Clinical Perspectives on Targeting of Myeloid Derived Suppressor Cells in the Treatment of Cancer

**DOI:** 10.3389/fonc.2013.00049

**Published:** 2013-03-15

**Authors:** Yana G. Najjar, James H. Finke

**Affiliations:** ^1^Department of Internal Medicine, The Cleveland Clinic FoundationCleveland, OH, USA; ^2^Department of Immunology, The Cleveland Clinic FoundationCleveland, OH, USA

**Keywords:** MDSC, targeted therapy, combination therapy, cancer, immune evasion

## Abstract

Tumors escape immune recognition by several mechanisms, and induction of myeloid derived suppressor cells (MDSC) is thought to play a major role in tumor mediated immune evasion. MDSC arise from myeloid progenitor cells that do not differentiate into mature dendritic cells, granulocytes, or macrophages, and are characterized by the ability to suppress T cell and natural killer cell function. They are increased in patients with cancer including renal cell carcinoma (RCC), and their levels have been shown to correlate with prognosis and overall survival. Multiple methods of inhibiting MDSCs are currently under investigation. These can broadly be categorized into methods that (a) promote differentiation of MDSC into mature, non-suppressive cells (all trans retinoic acid, vitamin D), (b) decrease MDSC levels (sunitinib, gemcitabine, 5-FU, CDDO-Me), or (c) functionally inhibit MDSC (PDE-5 inhibitors, cyclooxygenase 2 inhibitors). Recently, several pre-clinical tumor models of combination therapy involving sunitinib plus vaccines and/or adoptive therapy have shown promise in MDSC inhibition and improved outcomes in the tumor bearing host. Current clinical trials are underway in RCC patients to assess not only the impact on clinical outcome, but how this combination can enhance anti-tumor immunity and reduce immune suppression. Decreasing immune suppression by MDSC in the cancer host may improve outcomes and prolong survival in this patient population.

## Introduction

While several cancer treatments have been shown to illicit antigen specific immune responses, this has not correlated well with a clinical response and tumor regression. Multiple pre-clinical models have demonstrated regression of bulky tumors with immunotherapy, but the clinical response rates of several so called immunogenic tumors, including melanoma and renal cell carcinoma (RCC), remain quite low. It is widely accepted that the tumor microenvironment is immunosuppressive, both inhibiting activated immune cells and activating cells with a suppressive phenotype. Multiple cell types contribute to tumor mediated immune suppression, including regulatory T cells (T_reg_), type 2 NKT cells, tumor associated macrophages (TAMs), and myeloid derived suppressor cells (MDSCs). MDSCs are a heterogeneous cell population characterized by the ability to suppress T cell and natural killer (NK) cell function (Gabrilovich and Nagaraj, 2009; Ostrand-Rosenberg, 2010). They arise from myeloid progenitor cells that do not differentiate into mature dendritic cells, granulocytes, or macrophages. MDSCs have been shown to be significantly increased in cancer patients of all stages relative to healthy volunteers, with a significant correlation between circulating MDSC, metastatic burden, and clinical cancer stage (Diaz-Montero et al., 2009), and therefore offer an exciting new target in cancer therapy. The goal of this review is to summarize the rationale of therapeutic targeting of MDSC numbers and/or function in patients with cancer. This includes a discussion of MDSC subpopulations, particularly those in human cancer patients, along with a very brief description of the mechanisms used by MDSC to suppress T cell function, as this topic has been extensively reviewed by others (Gabrilovich et al., 2012). Included is a discussion of the various approaches used to reduce the number or function of MDSC, along with a summary of pre-clinical studies that have examined the impact of combining immunotherapy with approaches to reduce MDSC as a means to promote anti-tumor T cell immunity and decrease tumor progression.

### A heterogeneous population of MDSCs is induced by tumor mediated inflammation

Two main subsets are described in mouse tumor models, granulocytic, and monocytic. Granulocytic (G) MDSC are polymorphonuclear-like and account for 70–80% of the MDSC population (Movahedi et al., 2008; Youn et al., 2008), whereas monocytic (M) MDSCs are mononuclear and account for 20–30% of MDSCs (Youn et al., 2008). Identification of MDSC subsets in humans is more complex, with multiple populations defined in solid tumors, but are broadly defined as myeloid cells expressing CD33, CD11b, and low/negative HLA-DR. In general granulocytic and monocytic subsets represent major components of human MDSC and there may be subpopulations of each based on the markers used to define them. Additionally, MDSC with the phenotype of CD33+HLA-DR−/low that are linage negative (CD15−, CD14−) have also been well documented in cancer patients (Gabrilovich et al., 2012). The granulocytic subset expresses CD15 and/or CD66 and are typically negative for CD14 (Serafini et al., 2006a; Gabrilovich and Nagaraj, 2009; Ostrand-Rosenberg, 2010; Gabrilovich et al., 2012). For some types of human cancers such as RCC, granulocytic-MDSC with immunosuppressive activity is the prevalent population in the blood, although M-MDSC, linage negative (CD15−CD14−), and other subsets are also present (Zea et al., 2005; Kusmartsev et al., 2008; van Cruijsen et al., 2008; Rodriguez et al., 2009; Ko et al., 2010; Walter et al., 2012). Similar findings have been reported in glioma and bladder cancer patients (Raychaudhuri et al., 2011; Sippel et al., 2011). While a recent study in murine tumor models demonstrates that G-MDSC are functionally distinct from neutrophils and represent immature neutrophils with suppressive activity (Youn et al., 2012), the relationship between G-MDSC and neutrophils is less clear in human cancer patients. Cells with the phenotype of activated neutrophils have been shown to co-purify with peripheral blood mononuclear cells (PBMC) and MDSC during ficoll density centrifugation (Schmielau and Finn, 2001; Zea et al., 2005; Ko et al., 2009; Rodriguez et al., 2009) and are immunosuppressive, unlike neutrophils from healthy donors. Additionally, when neutrophils from healthy donors are activated they display prolonged survival, have reduced density, and are immunosuppressive, similar to MDSC (Schmielau and Finn, 2001; Rodriguez et al., 2009; Sippel et al., 2011). Moreover, immature neutrophils (CD66b+CD16−) also co-purify with PBMC (Brandau et al., 2011), although the suppressive activity of these cells is not well defined. It seems likely that activated neutrophils and immature granulocytes (G-MDSC) contribute to immune suppression in different types of human cancers, although the specific suppressive and angiogenic activity of these two cell types requires further study. The monocytic MDSC population is also present in many different tumor types and is typically CD14+HLA-DR−/low. In patients with melanoma, multiple myeloma, prostate, and hepatocellular carcinoma, the immuosuppressive M-MDSC is a prominent population (Filipazzi et al., 2007, 2012; Hoechst et al., 2008; Mandruzzato et al., 2009; Poschke et al., 2010; Vuk-Pavlovic et al., 2010) and is thought to suppress via the production of arginase, iNOS, and suppressive cytokines.

Myeloid derived suppressor cells are induced by chronic inflammation, and several tumor-secreted factors have been implicated in MDSC induction. Prostaglandin E2 induces differentiation of c-kit+ hematopoietic stem cells into MDSCs, contributing to T cell immune suppression (Rodriguez et al., 2005; Sinha et al., 2007b). Interleukin (IL)-6, IL-1β, GM-CSF, and G-CSF, which are found in the microenvironment of many tumors, have been shown to significantly increase MDSC accumulation and T cell suppression (Song et al., 2005; Bunt et al., 2006; Sinha et al., 2008). Furthermore, IL-1β induced inflammation aids MDSC and macrophage cross-talk, thus increasing MDSC mediated innate immune suppression (Bunt et al., 2006). In addition, proteins S100A8/A9, both pro-inflammatory, induce MDSC accumulation (Sinha et al., 2008). An autocrine positive feedback loop is created by MDSC secreting pro-inflammatory factors, including IL-6 and S100A8/A9, thus further sustaining themselves in the tumor microenvironment (Sinha et al., 2008; Ostrand-Rosenberg, 2010).

### MDSC are increased in peripheral blood and tumor parenchyma of the tumor host, and levels have been shown to correlate with clinical outcome

Several studies have shown increased MDSC levels in patients with different histologic tumors (Hoechst et al., 2008; Movahedi et al., 2008; Gabrilovich and Nagaraj, 2009). In a study of 106 patients with newly diagnosed stage I-IV solid tumors, circulating MDSC percentages were measured (Lin^−^/Low, HLA-DR^−^, CD33^+^CD11b^+^) prior to the start of treatment. Circulating MDSC levels were found to correlate both with clinical stage (*p* < 0.0001) and metastatic burden (*p* < 0.01). Interestingly, patients with radiographic evidence of disease progression had increased levels of circulating MDSC, whereas patients who responded to treatment had decreased MDSC (Diaz-Montero et al., 2009). A recent study identified six MDSC phenotypes using single multicolor staining: increased percentages of MDSC2–MDSC6 phenotypes were noted in patients with RCC compared to healthy donor controls (*p* < 0.01). Furthermore, a retrospective analysis found MDSC4 (monocytic; *p* < 0.001) and MDSC5 (granulocytic; *p* = 0.016) were significantly negatively associated with overall survival (Walter et al., 2012). Recently, increased circulating promyelocyte-like bone marrow derived CD11b^+^/CD16^−^MDSC levels correlated with reduced survival in breast cancer (*p* = 0.048) and colorectal cancer patients (*p* = 0.025) (Solito et al., 2011). Additionally, increased levels of HLA-DR Lin1^low/−^ CD33^+^ CD11b^+^ MDSC in pancreatic, esophageal, and gastric cancer was an independent prognostic factor for survival (*p* < 0.001) (Gabitass et al., 2011).

The presence of MDSC in hematological malignancies is not as well established, but they have been described in patients with multiple myeloma, Hodgkin’s lymphoma (HL), and non-Hodgkin’s lymphoma (NHL) (Montero et al., 2012). In the latter two, MDSC levels were found to correlate with clinical stage, and in NHL also correlated with faster rates of disease progression and more aggressive NHL histology (*p* = 0.01) (Motzer et al., 2002; Montero et al., 2012). Collectively, these early clinical findings suggest that accumulation of MDSC levels in cancer patients contributes to tumor progression, thereby providing a target for improving immunotherapy.

### MDSCs use various mechanisms to suppress effective anti-tumor immunity

**Figure 1:**
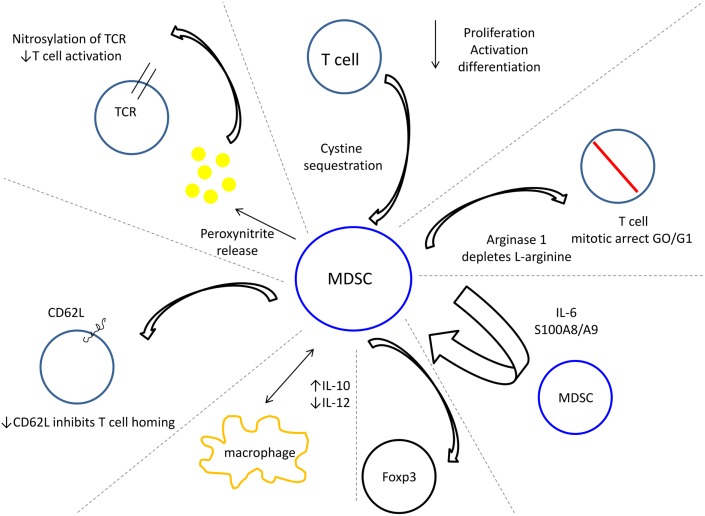
**Myeloid derived suppressor cells use multiple mechanisms to dampen anti-tumor immunity**.

The regulatory function of MDSC in dampening anti-tumor immunity has been extensively shown in both *in vitro* and *in vivo* studies (Figure 1). MDSC inhibit both antigen specific and non-specific T cell activation in murine MDSC co-cultures with peptide activated T cells and murine and human MDSC co-cultures with anti-CD3 activated T cells (Gabrilovich et al., 2001; Sinha et al., 2005). Both CD4^+^ and CD8^+^ T cells are suppressed, and while suppression requires cell to cell contact, this can occur by an MHC restricted or unrestricted mechanism (Nagaraj et al., 2007). Granulocytic and monocytic MDSC inhibit T cells by depletion of l-arginine within the tumor microenvironment, thus arresting T cells in G0–G1 (Rodriguez et al., 2005; Ostrand-Rosenberg, 2010). Similarly, MDSC inhibit T cell activation by sequestering cystine. This disables T cells from obtaining cysteine, which is essential for antigen activation, proliferation, and differentiation (Ostrand-Rosenberg, 2010; Srivastava et al., 2010). Reduced CD4^+^ and CD8^+^ T cell homing to lymph nodes is effected by MDSC, resulting in a down-regulation of L-selectin, which normally drives leukocyte extravasation to areas of inflammation (Hanson et al., 2009). MDSC have also been shown to impair innate immunity by their cross-talk with macrophages, which increases MDSC production of IL-10 and decreases macrophage production of IL-12, converting anti-tumor M1 cells into M2 cells that enhance tumor progression (Sinha et al., 2007a). In a murine B-cell lymphoma model, MDSC were identified as tolerogenic antigen presenting cells (APC) capable of antigen uptake and presentation to tumor-specific T_regs_ by an arginase dependant mechanism (Serafini et al., 2008). Interestingly, *in vitro* and *in vivo* inhibition of MDSC function reduced T_reg_ proliferation and tumor-induced tolerance in antigen specific T cells (Serafini et al., 2008).

## Different Strategies to Down-Regulate MDSC Number and Function

Given the phenotypic and functional heterogeneity of MDSCs, therapeutic approaches that are sufficient to inhibit MDSCs across a wide spectrum of cancer patients would be a significant addition to the anti-cancer armamentarium, and several mechanisms are currently undergoing investigation (Table 1).

**Table 1:** **Summary of mechanisms of anti-MDSC agents and key study findings**.

Mechanism of action	Agent	Study finding	Reference
Promoting MDSC differentiation	ATRA	Induced MDSC differentiation into myeloid DC in mice/humans. Improved T cell response	Gabrilovich et al. (2001)
		High plasma concentrations of ATRA correlated with reduced MDSC levels	Mirza et al. (2006)
	Vit D3	HNSCC pts treated with oral VitD3 had decreased MDSC	Lathers et al. (2004), Ugel et al. (2009)
Decreasing MDSC levels	Sunitinib	Treatment in RCC pts decreased MDSC (monocytic and linage negative subsets) and increased myeloid DC in patients experiencing tumor regression	van Cruijsen et al. (2008)
		Treatment in RCC pts decreased MDSC and T_reg_ levels, improved T cell function (IFNY production)	Finke et al. (2008), Ko et al. (2009)
		In mouse model (MCA 26) sunitinib reduced MDSC levels in tumor and also T_regs_ Synergized with immunotherapy to reduce tumor size Reduced PDL-1 expression	Ozao-Choy et al. (2009)
		In B16 Ova mouse model sunitinib reduced MDSC and T_eg_ in tumor Reduced levels of immunosuppressive co-stimulatory molecules and chemokines involved in MDSC and T_reg_ trafficking Synergy with vaccine to boost T cell anti-tumor response	Bose et al. (2010)
		Sunitinib reduced the viability of granulocytic-MDSC in tumor bearing mice and reduced the proliferation of monocytic MDSC	Ko et al. (2010)
	Axitinib	Reduced MDSC, T_reg_, and enhanced T cell response in tumor bearing mice	Bose et al. (2010)
	Gemcitabine	Decreased splenic MDSC, improved CD8 and NK cell anti-tumor activity in 5 murine lung cancer models Reduces number *ex vivo* and then they show apoptosis of splenocytes *in vivo*	Suzuki et al. (2005)
		Early treatment in a murine mammary carcinoma model decreased MDSC, which correlated with tumor growth inhibition	Le et al. (2009)
	5-FU	Treatment decreased splenic and intra tumor MDSC, did not affect T, B, NK, or dendritic cells. 5-FU triggers MDSC apoptosis	Vincent et al. (2010)
	Docetaxel	Reduced MDSC in spleen; increased CTL response; and polarized MDSC to M1 phenotype	Kodumudi et al. (2010)
Inhibiting MDSC function	CDDO-Me	In a murine model, decreased MDSC inhibitory function and decreased tumor growth. In RCC patients, completely abrogated MDSC inhibitory function *in vitro*	Nagaraj et al. (2010)
		In mice, did not affect number of MDSC in spleen, but eliminated suppressive activity of MDSC on CD8^+^ T cells *in vitro*	
Inhibiting MDSC function	PDE-5 inhibitor	In mice, treatment down regulated ARG1 and NOS2, abrogated suppressive pathways. In isolated cells from cancer patients, restored T cell proliferation	Serafini et al. (2006b)
		In melanoma patients, treatment decreased MDSC levels and weakened suppressive function	Umansky and Sevko (2012)
		Sildafenil increased survival of tumor bearing mice by a CD8^+^ T cell dependant mechanism. Decreased MDSC number and immunosuppressive function	Meyer et al. (2011)
	COX-2 inhibitor	In a murine glioma model, treatment inhibited PGE-2 production and delayed glioma development. MDSC were decreased in bone marrow and within the tumor, CCL2 chemokine was decreased also	Fujita et al. (2011)
		In ovarian cancer pts, decreased MDSC levels in ascites correlated with CXCL12 and PGE-2 inhibition	Obermajer et al. (2011)
	Nitro aspirin	Increased the number and function of tumor Ag-specific T lymphocytes *in vitro* and *in vivo* by decreasing ARG and NOS activity in CD11^+^ B lymphocytes	De Santo et al. (2005)

### Promoting differentiation of MDSC into mature, non-suppressive cells (ATRA, Vit D3)

Promoting differentiation of suppressive MDSC into mature, non-suppressive cells has been studied in pre-clinical and clinical cancer models, the rationale being that conversion of MDSC may enhance anti-tumor immune responses. Increased production of reactive oxygen species (ROS) is a functional characteristic of MDSC, and all trans retinoic acid (ATRA), a derivative of vitamin A, has been shown to induce MDSC differentiation by a glutathione synthase dependant mechanism (Nefedova et al., 2007). While ATRA induced differentiation of MDSC into myeloid dendritic cells *in vitro* (Gabrilovich et al., 2001), administration *in vivo* increased MDSC differentiation and enhanced CD4^+^ and CD8^+^ T cell antigen specific immune responses, but did not decrease tumor burden (Gabrilovich and Nagaraj, 2009). More promising results were obtained by combining ATRA with antigen specific peptide vaccines. In two different tumor models, treatment with ATRA and peptide vaccines significantly prolonged the anti-tumor treatment effect, making this molecule a promising candidate as an adjunct to cancer immunotherapy (Gabrilovich et al., 2001). The effect of ATRA on MDSC in cancer patients was recently elucidated: 18 patients with metastatic renal cell carcinoma (mRCC) who were shown to have elevated MDSC levels were treated with ATRA. This significantly reduced the number of MDSC in patients with a high plasma concentration of ATRA (>150 ng/mL), but not in patients with lower ATRA concentrations (<135 ng/mL) (Mirza et al., 2006). Interestingly, the effect of ATRA was abrogated in patients who also received subcutaneous IL-2 (Mirza et al., 2006).

In a phase IB study, treatment with oral Vit D3 in patients with HNSCC was shown to reduce the number of immune suppressive CD34^+^ cells (CD11b+CD33+CD14−HLA-DR−), increase HLA-DR expression, and increase plasma IL-12 and IFN-gamma levels *in vitro*, which would favor an anti-tumor Th1 immune response (Lathers et al., 2004; Ugel et al., 2009).

### Decreasing MDSC levels (sunitinib, gemcitabine, 5-FU)

Sunitinib is an oral receptor tyrosine kinase inhibitor that targets signaling by PDGFRs, VEGFRs, and c-kit, and was approved by the FDA for the treatment of advanced RCC in 2007, following a phase III trial that demonstrated improved overall and progression free survival (Motzer et al., 2009). It is currently front line therapy for patients with metastatic RCC. In patients with advanced RCC, after 4 weeks of sunitinib treatment, a generalized decrease in myeloid frequencies was observed (van Cruijsen et al., 2008). Increased levels of myeloid DC subsets were noted relative to other myeloid subsets in patients experiencing tumor regression, and high levels of CD1c/BDCA-1(+) MDSC were predictive of tumor regression and improved progression free survival (van Cruijsen et al., 2008), suggesting that sunitinib may play an immunomodulatory role in the tumor bearing host. In RCC patients, one cycle of treatment with sunitinib significantly increased the percentage of IFN-gamma producing T cells, reduced IL-4 production, and diminished type 2 bias (Finke et al., 2008). This augmented T cell response was associated with decreased MDSC levels, including a reduction in the dominant population, granulocytic-MDSC (Ko et al., 2009). The increase in type-1 response may be partly related to modulation of T_reg_ cells: mRCC patients were found to have a significantly higher number of T_reg_ than healthy controls, and while an inverse correlation between the increase in type-1 and a decrease in the percentage of T_reg_ was noted, the reduction in T_reg_ after treatment did not reach statistical significance (Finke et al., 2008). Additional studies in a mouse tumor model (4T1) indicate that sunitinib treatment may function by reducing the expansion of monocytic MDSC while inducing apoptosis in the granulocytic-MDSC subset (Ko et al., 2010). In an advanced tumor murine model, sunitinib treatment decreased both MDSC and T_reg_ levels, in addition to reducing suppressive function of MDSCs and improving tumor-specific T cell function (Ozao-Choy et al., 2009). Treatment with sunitinib also resulted in reduced expression of IL-10, transforming growth factor-beta, and Foxp3, but increased expression of IFN-gamma, skewing the immune response toward a Th1 phenotype, and increased cytotoxic T lymphocyte (CTL) responses in isolated tumor infiltrating lymphocytes (TILs). Perhaps most importantly, the expression of negative co-stimulatory molecules was widely dampened: CTLA4 and PD-1 were decreased in CD4^+^ and CD8^+^ T cells, and PDL-1 expression on MDSC and plasmacytoid dendritic cells was also significantly decreased by sunitinib treatment (Ozao-Choy et al., 2009).

STAT3 plays a central role in MDSC function, promoting tumor invasion, and angiogenesis. There is some evidence that sunitinib may act through a STAT3 associated mechanism. In a murine kidney cancer model (RENCA), sunitinib inhibited STAT3 activity in tumor associated MDSCs, and was found to reduce the expression of several STAT3 regulated pro-angiogenic genes (Kujawski et al., 2008; Xin et al., 2009).

While some chemotherapeutic agents, such as doxorubicin and cyclophosphamide, have been shown to increase MDSC levels in peripheral blood (Suzuki et al., 2005), gemcitabine, a cytidine nucleoside analog, has been shown to decrease splenic MDSC in murine models of five advanced lung cancer cell lines (Suzuki et al., 2005). Interestingly, no significant reduction was noted in CD4^+^ T cells, CD8^+^ T cells or B cells, and an increase in the anti-tumor activity of CD8^+^ T cells and activated NK cells was noted, making this a promising MDSC targeting agent. Furthermore, at specific time points after treatment, gemcitabine was shown to selectively induce MDSC apoptosis (Suzuki et al., 2005). In a more recent study, BALB/c mice inoculated with 4T1 mammary carcinoma were treated with repeated gemcitabine starting within 1 week after inoculation, or treated once after 20–25 days (Le et al., 2009). Early treatment with gemcitabine significantly decreased the proportion of MDSC in the spleen, and this correlated with a decrease in tumor growth (Le et al., 2009). While a single dose of gemcitabine in mice with large tumors did inhibit MDSC accumulation, this did not affect tumor burden. This study also suggests selective inhibition of MDSC, as gemcitabine treatment of tumor bearing mice restored CD8^+^ T cell immune function (Le et al., 2009).

5-FU, a pyrimidine analog, is another chemotherapeutic agent that has shown selective anti-MDSC activity. Treatment of tumor bearing mice with 5-FU led to a major decrease in splenic MDSC and MDSC within the tumor parenchyma, with no significant effect on T cells, B cells, NK cells, or dendritic cells (Vincent et al., 2010). Compared to gemcitabine, 5-FU showed more efficacy in MDSC depletion and induction of MDSC apoptotic cell death, both *in vitro* and *in vivo* (Vincent et al., 2010). Furthermore, 5-FU mediated elimination of MDSC increased IFN-gamma production by tumor-specific CD8^+^ T cells infiltrating the tumor, promoting T cell-dependent anti-tumor responses *in vivo* (Vincent et al., 2010).

### Functional inhibition of MDSC (PDE-5 inhibitors, COX-2 inhibitors, CDDO-Me)

PDE-5 inhibitors are currently widely used for the treatment of erectile dysfunction and pulmonary hypertension. Recently, multiple studies have elucidated their potential as anti-MDSC agents in cancer treatment. *In vitro*, PDE-5 inhibitors have been shown to have pro-apoptotic activity on chronic lymphocytic leukemia and colon carcinoma (Ugel et al., 2009). Experiments in immune deficient mice have clearly shown that this drugs’ anti-tumor effects are immune mediated. In multiple murine tumor models, several PDE-5 inhibitors were shown to synergize with adoptive cell therapy, delaying tumor growth (Serafini et al., 2006b). Furthermore, mice treated with PDE-5 inhibitor had increased CD8^+^ T cell intra tumor infiltration, and these lymphocytes up-regulated CD69 and CD25 (markers of activation) and secreted IL-2 (Serafini et al., 2006b). Most importantly, MDSC suppressive pathways were dampened: ARG1 and NOS2 were down regulated, in addition to IL-4-Rα expression (Serafini et al., 2006b). This strategy was also shown to be effective in cancer patients: in PBMC isolated from patients with head and neck cancer or multiple myeloma, PDE-5 inhibitors restored T cell proliferation (Serafini et al., 2006b).

More recently, studies have assessed the role of PDE-5 in melanoma. MDSC were found to be increased in melanoma lesions, and their accumulation was associated with a strong TCR ζ-chain down-regulation in T cells (Umansky and Sevko, 2012). Treatment with PDE-5 inhibitor resulted in decreased MDSC levels and partial restoration of ζ-chain expression in T cells, resulting in attenuated immunosuppressive function and significantly increased survival of tumor bearing mice, by a CD8^+^ T cell dependant mechanism (Meyer et al., 2011; Umansky and Sevko, 2012). These studies suggest that PDE-5 may be of benefit if used in conjunction with melanoma targeted immunotherapies.

The enzyme cyclooxygenase 2 (COX-2) plays a role in the production of PGE-2, which induces expansion of MDSC (Sinha et al., 2007b). In a murine glioma model, treatment with COX-2 inhibitors inhibited systemic PGE-2 production and delayed glioma development (Fujita et al., 2011). CCL2, an MDSC-attracting chemokine, was reduced in the tumor microenvironment, and MDSC were decreased both in the bone marrow and the tumor microenvironment (Fujita et al., 2011). Furthermore, increased levels of CTLs were noted in the tumor microenvironment (Fujita et al., 2011). These results were not observed in glioma-bearing COX-2 and CCL2 deficient mice (Fujita et al., 2011).

In a recent study, it was shown that PGE-2 attracts MDSC into the ascites microenvironment of ovarian cancer patients by inducing expression of functional CXCR4 in cancer-associated MDSCs, and plays a role in the production of its ligand CXCL12, thus ensuring MDSC migration (Obermajer et al., 2011). Frequencies of MDSCs closely correlated with CXCL12 and PGE-2 levels in ascitic fluid, and inhibition of COX-2 or PGE-2 receptors in MDSCs suppressed CXCR4 expression, and thus MDSC responsiveness to CXCL12 or ovarian cancer ascites (Obermajer et al., 2011). These studies provide a rationale for targeting COX-2 in cancer therapy.

CDDO-Me belongs to a class of relatively new compounds called synthetic triterpenoids, and has been shown to be a potent activator of the transcription factor NFR2, which up-regulates several antioxidant genes (Nagaraj et al., 2010). *In vitro*, CDDO-Me completely abrogated MDSC immunosuppressive activity from tumor bearing mice (Nagaraj et al., 2010), which is not surprising given that up-regulation of ROS is an essential function of MDSC. Treatment of mice with this agent did not decrease the proportion of splenic MDSC, but did eliminate MDSC suppressive activity, and decreased tumor growth (Nagaraj et al., 2010). Furthermore, CDDO-Me completely abrogated the inhibitory effect of MDSC *in vitro* in samples isolated from RCC patients (Nagaraj et al., 2010).

### Combination therapy: Targeting MDSC as an adjuvant to vaccines and immunotherapy

Current studies are focused on strategies that combine approaches to reduce MDSCs as an adjuvant to different forms of immunotherapy. As previously discussed, gemcitabine has been shown to reduce splenic MDSC levels in tumor bearing mice (Suzuki et al., 2005). In this same study, combining gemcitabine with IFN-beta markedly enhanced anti-tumor efficacy (Suzuki et al., 2005). In a HER-2/neu tumor model, treatment with gemcitabine, HER-2/neu vaccine and anti-glucocorticoid tumor necrosis factor receptor related protein (GITR) mAbs showed potent therapeutic anti-tumor immunity, in addition to protection against pre-existing tumors (Ko et al., 2007). Given that Her-2/neu is a self antigen with poor immunogenicity, this study suggests that when given with antigen specific immunotherapy, gemcitabine combinational therapy may be more effective than either treatment alone (Table 2).

**Table 2:** **Summary of combination therapies targeting MDSC and key study findings**.

Agents	Study finding	Reference
ATRA + antigen specific peptide vaccine	In two different murine tumor models, significantly prolonged the anti-tumor treatment effect	Gabrilovich et al. (2001)
Gemcitabine + IFN-b	Significantly increased anti-tumor activity in a murine tumor model	Suzuki et al. (2005)
Gemcitabine + HER-2/neu vaccine + anti-GITR mAb	Potent therapeutic anti-tumor immunity in a murine tumor model	Ko et al. (2007)
Sunitinib + low-dose radiotherapy	Modestly improved survival in a mouse glioma model Sunitinib with high dose radiation resulted in fatal toxicities	D’Amico et al. (2012)
Sunitinib + DC based vaccine	Combination Rx had superior anti-tumor effect than either Rx alone in a murine melanoma model and enhanced anti-tumor T cell response and reduced MDSC/T_reg_	Bose et al. (2010)
Sunitinib + adoptive T cell therapy	In murine melanoma and RCC models Inhibited Stat3 in DC and T cells Reduced conversion of T cells to T_regs_ Increased CD8^+^ T cell infiltration and activation at the tumor site Inhibited primary tumor growth	Kujawski et al. (2010)
Sunitinib + IL-12 + 4-1BB activation	Significantly improved long-term survival rate of large tumor bearing mice in liver and lung tumor models, promoted T cell response and reduced MDSC levels	Ozao-Choy et al. (2009)
Sunitinib + CEA vaccine	In a murine colon cancer model: continuous sunitinib followed by vaccine increased tumor infiltration of Ag-specific T lymphocytes Reduced tumor volumes Increased survival Decreased T_reg_ Decreased MDSC	Farsaci et al. (2012)
Bevacizumab ± IL-2	Did not reduce MDSC levels in the peripheral blood	Rodriguez et al. (2009)
Phase III trial, TroVAax (MVA-5T4) vaccine + sunitinib, IL-2, or IFN-a	In RCC pts did not enhance survival relative to sunitinib, IL-2, or IFN-a alone	Amato et al. (2010)

Several studies have shown that tumor-directed radiation therapy increases the effectiveness of several forms of immunotherapy (Kao et al., 2011). While the exact mechanism has yet to be elucidated, this may be due to increased uptake of tumor antigen by APCs within the irradiated field. In a recent mouse glioma model, addition of sunitinib to low-dose radiotherapy only modestly improved survival (D’Amico et al., 2012). Combining sunitinib with high dose radiation therapy resulted in fatal toxicities, though each treatment was well tolerated alone, thus limiting the feasibility of this combination (D’Amico et al., 2012). Unfortunately, success with the combination of sunitinib and radiation has been on a case by case basis, with no clinical series to date assessing the potential synergy of this combination (Dallas et al., 2012; Venton et al., 2012).

In patients with RCC, mutation the VHL tumor suppressor gene results in overproduction of vascular endothelial growth factor (VEGF). Athymic nude mice that were inoculated with human RCC cells were found to have VEGF receptor 1 (VEGFR1)/CD11b myeloid cells in the peripheral blood (Kusmartsev et al., 2008). Treatment with Avastin (humanized anti-VEGF-1 mAb) resulted in significantly reduced numbers of circulating VEGFR1+ MDSC, suggesting that elimination of VEGFR1+ cells may restore immunocompetence (Kusmartsev et al., 2008). However, treatment of metastatic RCC patients with bevacizumab either alone or combined with interleukin-2 did not reduce MDSC levels in the peripheral blood (Rodriguez et al., 2009). The difference in MDSC modulation between these two studies may be related to the timing of antibody administration, since RCC patients had advanced disease while mice were treated with antibody during early stages of tumor development.

Recent animal models have suggested that inhibiting MDSC and thus reversing immune suppression with sunitinib, a tyrosine kinase inhibitor, may be an effective adjunctive treatment to immune-based cancer therapies (Ozao-Choy et al., 2009; Bose et al., 2010; Kujawski et al., 2010). However, in a phase III trial, combining the TroVAax (MVA-5T4) vaccine with either sunitinib, IL-2, or IFN-α in RCC patients did not enhance survival relative to sunitinib alone (or IL-2 or IFN-α alone) (Amato et al., 2010). Interestingly, the lack of synergy between vaccine and sunitinib in this trial may be related to the sequence of vaccine and sunitinib administration. In an MC38-CEA murine tumor model, treatment with sunitinib followed by vaccine was most effective compared to the reverse order, suggesting that in some tumor models the sequencing of sunitinib and vaccine is important (Farsaci et al., 2012). Further studies are needed to assess the role of combining sunitinib with immunotherapy in t he clinical setting. Indeed, two company supported clinical trials are underway to test the efficacy and immune modulating activity of combining sunitinib with vaccines in metastatic RCC patients (Argos Therapeutics and Immatics Biotechnologies).

## Conclusion

Immune evasion is a hallmark of cancer, and MDSC play a central role in tumor mediated immunosuppression. MDSC are increased in the tumor bearing host, and MDSC levels have been shown to correlate with disease stage and survival. Multiple studies show that targeting MDSC leads to an improvement in anti-tumor immunity, specifically recovery of CD8^+^ T cell anti-tumor activity, resulting in tumor suppression, and multiple modes of targeting MDSC are in clinical development. For example, administration of ATRA to patients with metastatic RCC increased MDSC differentiation and enhanced CD4^+^ and CD8^+^ T cell antigen specific immune responses (Gabrilovich and Nagaraj, 2009). In another study, treatment with oral Vit D3 in patients with HNSCC reduced the number of immune suppressive CD34^+^ cells and skewed immune system toward an anti-tumor Th1 immune response (Lathers et al., 2004; Ugel et al., 2009). However, while multiple studies have shown effective antigen specific immunity, this has not correlated with improved survival: reduction in immune suppression by MDSC may improve outcomes using cancer vaccines and other forms of immunotherapy.

## Conflict of Interest Statement

Yana G. Najjar declares no conflicts of interest. James H. Finke has received research grants from Pfizer and GSK.
